# Transcriptomic and Proteomic Analysis of Shaan2A Cytoplasmic Male Sterility and Its Maintainer Line in *Brassica napus*

**DOI:** 10.3389/fpls.2019.00252

**Published:** 2019-03-04

**Authors:** Luyun Ning, Hao Wang, Dianrong Li, Zhiwei Lin, Yonghong Li, Weiguo Zhao, Hongbo Chao, Liyun Miao, Maoteng Li

**Affiliations:** ^1^Department of Biotechnology, College of Life Science and Technology, Huazhong University of Science and Technology, Wuhan, China; ^2^Hybrid Rape Research Center of Shaanxi Province, Shaanxi Rapeseed Branch of National Centre for Oil Crops Genetic Improvement, Yangling, China; ^3^Hubei Collaborative Innovation Center for the Characteristic Resources Exploitation of Dabie Mountains, Huanggang Normal University, Huanggang, China

**Keywords:** cytoplasmic male sterility, transcriptome, proteome, transcription factor, anther differentiation, mitochondria, chloroplasts

## Abstract

Cytoplasmic male sterility (CMS) lines are widely used for hybrid production in *Brassica napus*. The Shaan2A CMS system is one of the most important in China and has been used for decades; however, the male sterility mechanism underlying Shaan2A CMS remains unknown. Here, we performed transcriptomic and proteomic analysis, combined with additional morphological observation, in the Shaan2A CMS. Sporogenous cells, endothecium, middle layer, and tapetum could not be clearly distinguished in Shaan2A anthers. Furthermore, Shaan2A anther chloroplasts contained fewer starch grains than those in Shaan2B (a near-isogenic line of Shaan2A), and the lamella structure of chloroplasts in Shaan2A anther wall cells was obviously aberrant. Transcriptomic analysis revealed differentially expressed genes (DEGs) mainly related to carbon metabolism, lipid and flavonoid metabolism, and the mitochondrial electron transport/ATP synthesis pathway. Proteomic results showed that differentially expressed proteins were mainly associated with carbohydrate metabolism, energy metabolism, and genetic information processing pathways. Importantly, nine gene ontology categories associated with anther and pollen development were enriched among down-regulated DEGs at the young bud (YB) stage, including microsporogenesis, sporopollenin biosynthetic process, and tapetal layer development. Additionally, 464 down-regulated transcription factor (TF) genes were identified at the YB stage, including some related to early anther differentiation such as *SPOROCYTELESS* (*SPL*, also named *NOZZLE*, *NZZ*), *DYSFUNCTIONAL TAPETUM 1* (*DYT1*), *MYB80* (formerly named *MYB103*), and *ABORTED MICROSPORES* (*AMS*). These results suggested that the sterility gene in the Shaan2A mitochondrion might suppress expression of these TF genes in the nucleus, affecting early anther development. Finally, we constructed an interaction network of candidate proteins based on integrative analysis. The present study provides new insights into the molecular mechanism of Shaan2A CMS in *B. napus*.

## Introduction

Rapeseed (*Brassica napus*) is an important oil crop producing both edible oil and industrial materials such as lubricants and biodiesel. Heterosis is widely observed in *B. napus*, with excellent hybrids having yields over 30% higher than their parents (Radoev et al., 2008). Hybrid breeding largely relies on male-sterile lines, mainly involving chemically induced male sterility (CIMS), genic male sterility (GMS), and cytoplasmic male sterility (CMS) (Chen and Liu, 2014). In higher plants, CMS is a maternally inherited trait, which is largely resulting from rearrangements of mitochondrial DNA, which results in an inability to generate pollen or in abnormal pollen (Schnable and Wise, 1998; Horn et al., 2014). The first CMS line was found in onion (Jones and Clarke, 1943). To date, CMS has been observed in more than 150 plant species (Laser and Larsten, 1972; Schnable and Wise, 1998). Four major CMS systems have been used in rapeseed production: *nap* CMS (Shiga and Baba, 1971), *pol* CMS (Fu, 1981), *Ogu* CMS (Ogura, 1968), and Shaan2A CMS (Li, 1980). *pol* CMS and Shaan2A CMS are the most commonly used CMS systems in *B. napus* in terms of number of three-line hybrids and the area planted with these hybrids (Fu, 1995).

In recent years, transcriptomic and proteomic analyses have been used in CMS studies. The transcriptome of CMS onion revealed three nuclear-related genes, *AGAMOUS* (*AG*), *ABORTED MICROSPORES* (*AMS*), and *SOMATIC EMBRYOGENESIS RECEPTOR-LIKE KINASE 1* (*SERK1*) (Yuan et al., 2018). Transcriptomic analysis in *Gossypium hirsutum* indicated decreased ability to eliminate reactive oxygen species (ROS) in the CMS line, with ROS released from mitochondria acting as signal molecules in the nucleus, triggering formation of abnormal tapetum (Suzuki et al., 2013; Yang et al., 2018). In *Ogu* CMS, key genes participating in the secretion and translocation of sporopollenin precursors were significantly down-regulated in the cabbage R2P2CMS line (Xing et al., 2018). Differentially expressed genes (DEGs) involved in protein synthesis and metabolic pathways have been identified between sterile and maintainer lines in both *pol* CMS and SaNa-1A CMS in *B. napus* (An et al., 2014; Du et al., 2016).

In flowering plants, anthers are important organs that generate pollen grains for propagation. In Arabidopsis, anther development has been systematically divided into 14 stages (Sander et al., 1999). First, the stamen primordium arises from the floral apex, then the archesporial cells from the four corners of the L2 cell layer undergo a series of differentiation and division events to promote formation of the four microsporangia of the butterfly shaped anther (Feng and Dickinson, 2007). These processes are controlled by a number of genes that function downstream of floral identity genes, most of them unknown. In Arabidopsis, *AG* has dual roles in limiting stem cell proliferation and determining floral organ identities (Lohmann et al., 2001). *SPL*, encoding a MADs transcription factor (TF), is a direct downstream gene of *AG* and the candidate gene for specifying the identity, position, and number of archesporial cells in the anther L2 layer; microsporogenous cells are not generated in the *spl* mutant (Schiefthaler et al., 1999; Yang et al., 1999). Another gene, *HYPONASTIC LEAVES1* (*HYL1*), modulates the stamen architecture of the four microsporangia by coordinating expression of *REVOLUTA* (*REV*) and *FILAMENTOUS FLOWER* (*FIL*) through *miR165/166* in Arabidopsis (Lian et al., 2013). Interestingly, *FIL* directly interacts with *SPL* (Sieber, 2004). Recently, *GROWTH-REGULATING FACTOR* (*GRF*) and *GRF-INTERACTING FACTOR* (*GIF*) were identified as novel positive regulators in specifying archesporial cells in Arabidopsis, and *grf1/2* and *gif1/2* mutants show a similar phenotype to the *spl* mutant (Lee et al., 2018). *BARELY ANY MERISTEM* (*BAM1* and *BAM2*) encode CLAVATA1-related and leucine-rich repeat (LRR) receptor-like kinases, and anthers in the double mutant *bam1 bam2* exhibit abnormalities at a very early stage, lacking the tapetum, middle layers, and endothecium (Hord, 2006). *EXCESS MICROSPOROCYTES 1* (*EMS1*, also known as *EXTRA SPOROGENOUS CELLS*, *EXS*) also encodes a putative LRR receptor kinase, regulating tapetum identity and number of reproductive cells in Arabidopsis (Canales et al., 2002; Zhao et al., 2002). Interestingly, *SPL* is expressed in most early anther development mutants, for example, *bam1/2* double mutants and *ems1* (Canales et al., 2002; Zhao et al., 2002; Hord, 2006), indicating that *SPL* might be the first “reproductive gene” to be activated in anther development.

Shaan2A CMS was identified by Professor Dianrong Li in 1976, and the representative hybrid rapeseed cultivar ‘Qinyou 2’ was generated using the Shaan2A CMS line and its restorer line KC01. This was the first three-line hybrid cultivar in China, and was widely cultivated from 1985 to 2008 (Li and Tian, 2015). Although the Shaan2A CMS system has been widely used in the production of hybrids for decades, its male sterility mechanism remains unclear. In the present study, we performed transcriptomic and proteomic analyses, combined with additional morphological observation, to reveal the mechanism of Shaan2A CMS. We aimed to identify differences between the sterile line Shaan2A and its maintainer line Shaan2B at the transcriptional and protein level, and elucidate the regulative and metabolic pathways involved in the male sterility. The results will provide new insights into the molecular mechanism of Shaan2A CMS in *B. napus*.

## Materials and Methods

### Plant Materials

Male sterile plants were found among hybrid offspring by Professor Dianrong Li in 1976. The male sterile lines were conserved by self-fertilization with limited pollen, hybridization of sister plants, and test crossing of different rapeseed cultivars. The male sterile Shaan2A line and its maintainer line Shaan2B were then rapidly bred by individual pollination of a single plant, biparental crossing, and mutual selection of parents according to the female parent’s phenotype as well as sterility of the female and agronomic characteristics of the parents. In the present study, Shaan2A and Shaan2B were cultivated in the test field of Huazhong University of Science and Technology (Wuhan, Hubei, China) under the same conditions. Plant samples were quickly removed from buds of different length on ice and frozen in liquid nitrogen, then kept at −80°C for extraction of total RNA and protein.

### Observation of Paraffin Sections and Transmission Electron Microscopy (TEM)

Anthers of different length were vacuum-infiltrated and fixed with 50%FAA (v/v) and 2.5% (w/v) glutaraldehyde in 0.1 M phosphate buffer (pH 7.4). For paraffin section analysis, fixed materials were dehydrated through a graded series of ethanol (70, 85, 90, 95, 2 × 100%) and then cleared twice with xylene for 2 h. The material was infiltrated with Paraffin wax and subsequently embedded in paraffin wax. Finally, sections of approximately 10 μm were obtained using a KD-1508A microtome (China) and observed under a LEICA DMLB microscope (Germany) with a CCD camera (DFC420 FX). Procedures for TEM observation followed a previous study (Yi et al., 2010) with minor modification (Ning et al., 2018).

### Proteomic Analysis

Different-sized anthers were collected from flower buds and mixed together. Preparation of total protein, two-dimensional electrophoresis (2-DE), analysis of differentially expressed proteins (DEPs), and protein identification by MALDI-TOF-MS-MS were conducted as described previously (Gan et al., 2013; Ning et al., 2018).

### Transcriptomic Analysis

*B. napus* anthers from buds <0.5, 0.5–1, 1–1.5, and 1.5–2.0 mm are at the pollen mother cell stage, meiosis stage, tetrad stage, and stage of microspore release from the tetrad, respectively (Zhou et al., 2012). Young buds (YB) shorter than 1 mm represented the stage before meiosis, and small anthers (SA) in 1–2 mm buds represented the tetrad stage to the microspore release stage. Total RNA extraction, assembly, and mapping of clean reads, and annotation of the transcriptome were conducted according to a previous report (Ning et al., 2018). For Kyoto Encyclopedia of Genes and Genomes (KEGG) analysis^1^, KOBAS software was used to test the statistical enrichment of DEGs in KEGG pathways.

### qRT-PCR

Total RNA was reverse-transcribed using ReverTra Ace^®^ qPCR-RT Master Mix with gDNA Remover (TOYOBO) according to the protocol of manufacturer, with *actin* as an internal reference gene (An et al., 2014). qRT-PCR experiments were conducted and calculations performed as described previously (Miao et al., 2016). All primer sequences are given in Supplementary Table S1.

### Interaction Analysis

Interaction analysis of DEGs and DEPs was based on the STRING database^2^, including known and predicted protein–protein interactions. Blastx (v2.2.28) was used to align target gene sequences to reference protein sequences, and a network was constructed according to known interactions in *Arabidopsis thaliana*.

## Results

### Morphological and Cytological Comparison Between Shaan2A and Shaan2B

**FIGURE 1:**
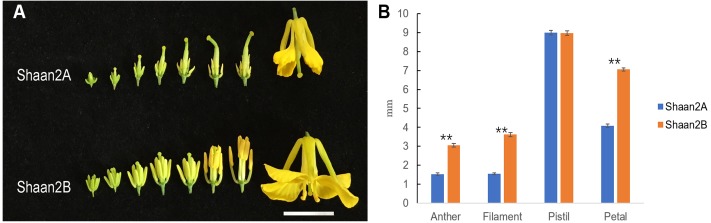
Phenotypic characterization of fertile and sterile buds. **(A)** Shaan2A and Shaan2B buds. Bar: 1 cm. **(B)** Length of anther, filament, and pistil, and width of petal in opened flowers (Student’s *t*-test,^∗∗^*P* < 0.01).

**FIGURE 2:**
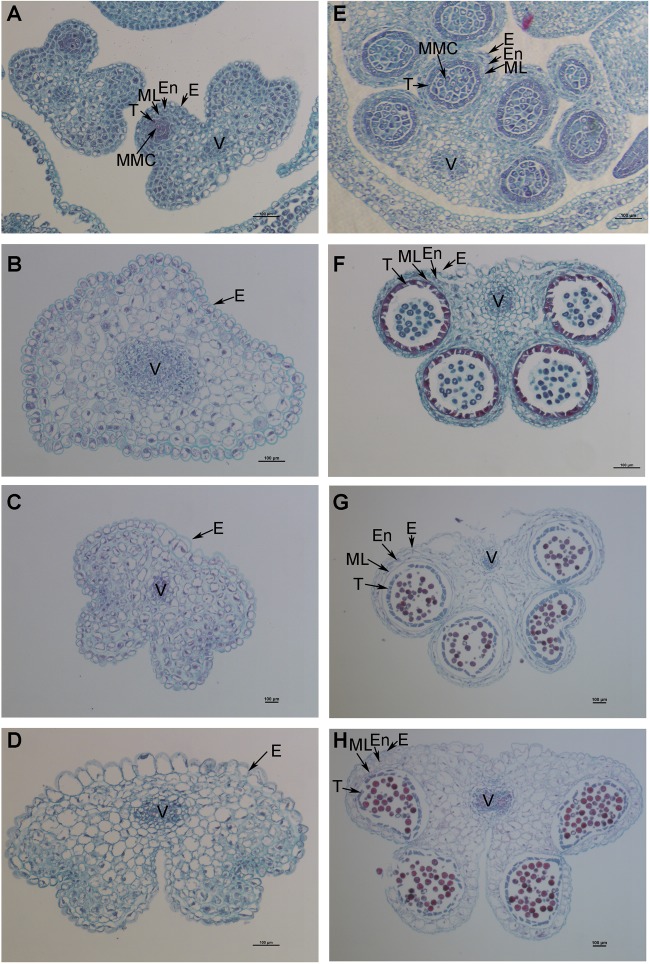
Dynamic observation of paraffin sections of Shaan2A and Shaan2B anthers. **(A)** Anthers in 1 mm buds of Shaan2A. **(B)** Anthers in 2 mm buds of Shaan2A. **(C)** Anthers in 3 mm buds of Shaan2A. **(D)** Anthers in 4 mm buds of Shaan2A. **(E)** Anthers in 1 mm buds of Shaan2B. **(F)** Anthers in 2 mm buds of Shaan2B. **(G)** Anthers in 3 mm buds of Shaan2B. **(H)** Anthers in 4 mm buds of Shaan2B. E, epidermis; En, endothecium; ML, middle layer; T, tapetum; MMC, microspore mother cells; V, vascular region. Bar: 100 μm.

The petals of Shaan2B flowers were significantly wider than those of Shaan2A flowers, and the anthers and filaments in Shaan2B were obviously longer than those in Shaan2A (Figure 1A,B). The anthers in Shaan2A appeared to stop developing at an early stage and hardly split, releasing pollen eventually, while the pistil in Shaan2B was normal. Cytological differences between the anthers of Shaan2A and Shaan2B are shown in Figure 2. The anthers in 1 mm buds of Shaan2B were developed normally, and it was easy to distinguish the sporogenous cells and the middle layer, endothecium, and tapetum (Figure 2E). However, most of the anther locules in 1 mm buds of Shaan2A were differentiated abnormally, and the anther wall and sporogenous cells could not be clearly distinguished (Figure 2A). In 2–4 mm buds, the anthers of Shaan2B were well developed, while the anthers in Shaan2A were obviously aberrant (Figure 2).

**FIGURE 3:**
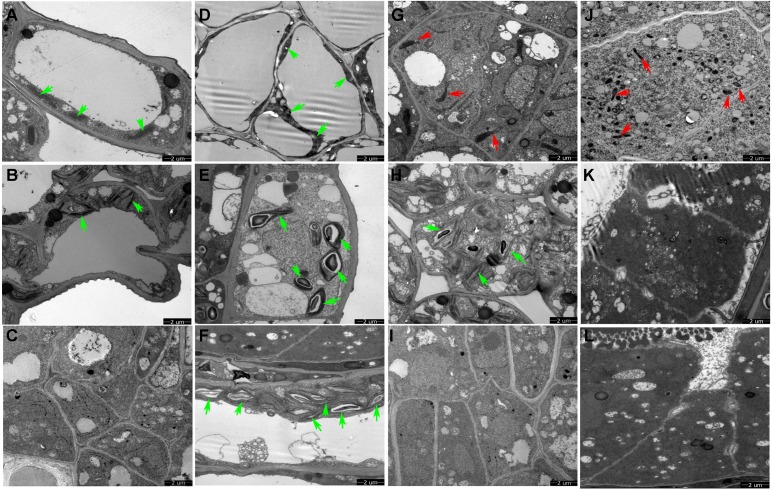
TEM observation of epidermis and tapetum cells of Shaan2A and Shaan2B anther walls. **(A)** Epidermis cells in 1 mm buds of Shaan2A. **(B)** Epidermis cells in 2 mm buds of Shaan2A. **(C)** Epidermis cells in 3 mm buds of Shaan2A. **(D)** Epidermis cells in 1 mm buds of Shaan2B. **(E)** Epidermis cells in 2 mm buds of Shaan2B. **(F)** Epidermis cells in 3 mm buds of Shaan2B. **(G)** Tapetum cells in 1 mm buds of Shaan2A. **(H)** Tapetum cells in 2 mm buds of Shaan2A. **(I)** Tapetum cells in 3 mm buds of Shaan2A. **(J)** Tapetum cells in 1 mm buds of Shaan2B. **(K)** Tapetum cells in 2 mm buds of Shaan2B. **(L)** Tapetum cells in 3 mm buds of Shaan2B. Green arrows indicate chloroplasts and red arrows represent mitochondria. Bar: 2 μm.

We conducted TEM observation to further understand ultrastructural differences in the anther wall between Shaan 2A and Shaan2B. As shown in Figure 3A, epidermis cells in 1 mm buds of Shaan2A had few starch grains in chloroplasts and a large vacuole. Except for the large vacuole, Shaan2B epidermis cells had starch grains in most of the chloroplasts (Figure 3D). Tapetum cells in 1 mm buds of Shaan2A had a few mitochondria (2.8 ± 0.98) (Figure 3G), while those in Shaan2B were enriched with mitochondria (19 ± 1.55) (Figure 3J). The lamella structure of chloroplasts in both epidermis and tapetum cells were obviously abnormal in 2 mm buds of Shaan2A (Figure 3B,H). However, lamella structure in Shaan2B was normal (Figure 3E), and the structure of tapetum cells in Shaan2B (Figure 3K) were distant not alike with those in Shaan2A (Figure 3H). Moreover, epidermis and tapetum cells in 3 mm buds of Shaan2A had the same structure (Figure 3C,I), while those of Shaan2B exhibited different differentiation (Figure 3F,L). The different cell layers in anther walls during early anther differentiation are roughly shown in Supplementary Figure S1. These phenomena indicated that the differentiation of anther wall cells in Shaan2A was disordered.

### Comparative Transcriptomic Analysis Between Shaan2A and Shaan2B

We used comparative transcriptomics to identify genes associated with male sterility in Shaan2A CMS. RNA-seq of three biological replicates at the YB (<1 mm buds, before meiosis) and SA (1–2 mm buds, representing tetrad to microspore release) stages generated 637,533,762 raw reads. The raw reads were deposited in the SRA (NCBI Sequence Read Archive) database under accession number PRJNA502996. These raw data were filtered to obtain clean reads, of which over 90% perfectly matched to the reference genome (Chalhoub et al., 2014). More detailed information for RNA-seq data is shown in Table 1.

**FIGURE 4:**
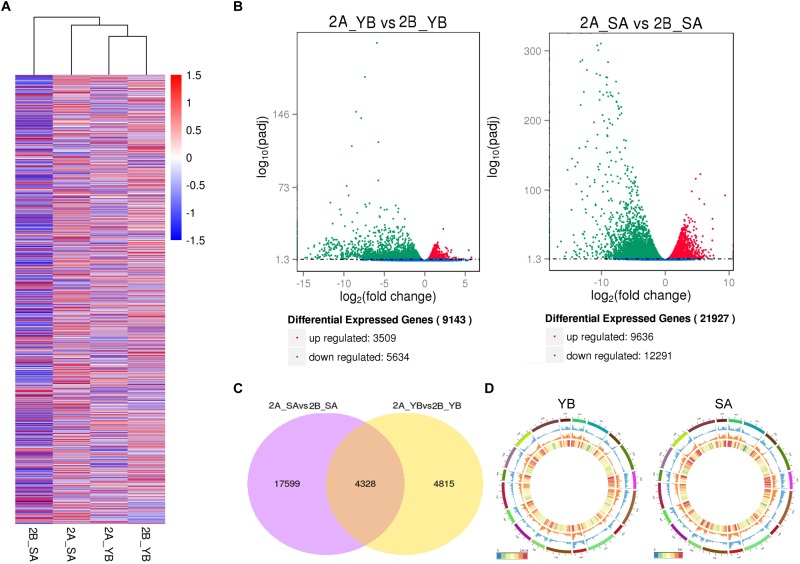
Statistical analysis of gene expression detected by transcriptomic analysis. **(A)** Heatmap analysis of all DEGs at the different stages. **(B)** Volcano plot of up- and down-regulated genes at the YB and SA stages. **(C)** Venn diagram showing common DEGs at the two stages. **(D)** Circle plot of the distribution of SNPs and indels, and the density of DEGs in different chromosomal regions. The outermost layer represents all *B. napus* chromosomes (A01–A10 and C01–C09). The blue layer represents the distribution of SNPs, the middle orange layer shows the distribution of indels, and the innermost layer indicates the density of DEGs.

We performed hierarchical cluster analysis of all DEGs to examine global gene expression patterns in different samples (Figure 4A). Shaan2A_YB and Shaan2B_YB showed similar global expression patterns. When compared with Shaan2B, a total of 3,509 and 9,636 up-regulated DEGs and 5,634 and 12,291 down-regulated DEGs (*P* < 0.05) were identified at the YB and SA stages in Shaan2A, respectively (Figure 4B). The number of DEGs shared at the two stages was 4,328, only 1/5 of the total DEGs at the SA stage (Figure 4C). The number of DEGs, and the densities of single-nucleotide polymorphisms (SNPs) and insertions and deletions (indels) in different chromosomal regions at the YB and SA stages, respectively, are shown in Figure 4D. High-density areas of both SNPs and indels were focused on chromosomes A01, A02, A03, and A05, and DEGs were more concentrated in genome A than genome C.

KEGG analysis (*P* < 0.05) of DEGs between Shaan2A and Shaan2B at the two stages revealed that pathways associated with protein processing in endoplasmic reticulum, carbon metabolism, and fatty acid degradation were enriched at the YB stage (Supplementary Table S2), as some genes in these pathways, such as *HEAT SHOCK PROTEIN* (*Hsp70*), *SHEPHERD* (*SHD*), and *LONG CHAIN ACYL-COA SYNTHETASE 9* (*LACS9*) were significantly differentially expressed. In Arabidopsis, *Hsp70* is a molecular chaperone that plays numerous important roles in protein folding (Wisén and Gestwicki, 2008). At the SA stage, genes associated with starch and sucrose metabolism, porphyrin and chlorophyll metabolism, and carbon metabolism pathways were annotated (Supplementary Table S2). Among these genes, *FERROCHELATASE (FC)* and *CHLOROPHYLLASE (CLH)* were identified. *FC* is the last enzyme for heme biosynthesis in the tetrapyrrole pathway and is essential for stress tolerance in Arabidopsis (Chow et al., 1998; Fan et al., 2018).

**Table 1:** Summary of RNA-seq data.

Sample	Replicate	Raw reads	Clean reads	GC content (mol%)	Total mapped	Multiply mapped	Uniquely mapped
2A_YB	2A_YB1	44,035,120	43605746	46.96	39,475,845 (90.53%)	1,639,764 (3.76%)	37,836,081 (86.77%)
	2A_YB2	45,983,586	45630640	46.85	41,183,271 (90.25%)	1,686,124 (3.70%)	39,497,147 (86.56%)
	2A_YB3	45,953,444	45231052	47.04	40,916,267 (90.46%)	1,715,471 (3.79%)	39,200,796 (86.67%)
2A_SA	2A_SA1	59,213,734	58019808	46.09	52,317,347 (90.17%)	2,184,243 (3.76%)	50,133,104 (86.41%)
	2A_SA2	59,374,138	58794086	46.2	53,061,323 (90.25%)	2,147,433 (3.65%)	50,913,890 (86.6%)
	2A_SA3	62,914,554	62064672	46.42	56,103,044 (90.39%)	2,325,942 (3.75%)	53,777,102 (86.65%)
2B_YB	2B_YB1	50,061,626	49538462	46.88	44,776,518 (90.39%)	1,864,618 (3.76%)	42,911,900 (86.62%)
	2B_YB2	62,070,224	61306156	46.82	55,684,014 (90.83%)	2,297,159 (3.75%)	53,386,855 (87.08%)
	2B_YB3	46,417,324	45834614	46.95	41,562,082 (90.68%)	1,664,307 (3.63%)	39,897,775 (87.05%)
2B_SA	2B_SA1	55,769,766	55185458	45.98	49,900,475 (90.42%)	2,448,037 (4.44%)	47,452,438 (85.99%)
	2B_SA2	52,729,352	51938752	46.21	47,032,360 (90.55%)	2,219,473 (4.27%)	44,812,887 (86.28%)
	2B_SA3	53,010,894	52211658	46.12	47,256,054 (90.51%)	2,307,822 (4.42%)	44,948,232 (86.09%)

**FIGURE 5:**
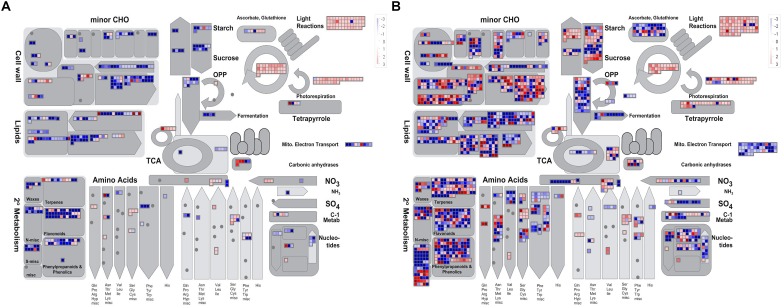
Overview of the metabolic pathways the DEGs were involved in at the YB and SA stages. **(A)** Overview of YB stage. **(B)** Overview of SA stage.

To obtain an overview of the pathways in which the DEGs participated, we further analyzed the DEGs (| log_2_ ratio| > 1) at the YB and SA stages using MapMan software. The metabolism pathway visualization is shown in Figure 5, and detailed information is listed in Supplementary Table S3. Although the DEGs were dispersed in different primary and secondary metabolism pathways, most involved in lipid and flavonoid metabolism and mitochondrial electron transport/ATP synthesis pathways were down-regulated at both the YB (Figure 5A) and SA (Figure 5B) stages; for example, *BnaA02g21480D*, which encodes a member of the SMO1 family of sterol 4-alpha-methyl oxidases in the lipid metabolism pathway, and *BnaA02g33800D*, encoding flavonol synthase 3 (*FLS3*) in the flavonoid metabolism pathway. Interestingly, a large number of DEGs were up-regulated in light reactions, photorespiration, and the Calvin cycle (Figure 5A,B). These included *BnaA03g22080D*, which encodes a PsbP domain-OEC23 like protein localized in thylakoids (peripheral-lumenal side) in the light reaction pathway, *BnaA01g00170D*, which encodes a protein with mitochondrial serine hydroxymethyl transferase activity and functions in the photorespiration pathway, and *BnaC04g05700D*, which encodes a Rubisco activase and functions in the Calvin cycle.

Furthermore, 11 DEGs related to anther development were selected for qRT-PCR validation (Supplementary Figure S2), for example, *BnaA01g09760D* (*AG*), *BnaA10g23720D* (*EMS1*), and *BnaA01g16350* (*SPL*). Most of the expression patterns of these DEGs at the two stages were basically consistent with the RNA-seq data, indicating that the RNA-seq data were reliable.

### Comparative Proteomic Analysis Between Shaan2A and Shaan2B

**FIGURE 6:**
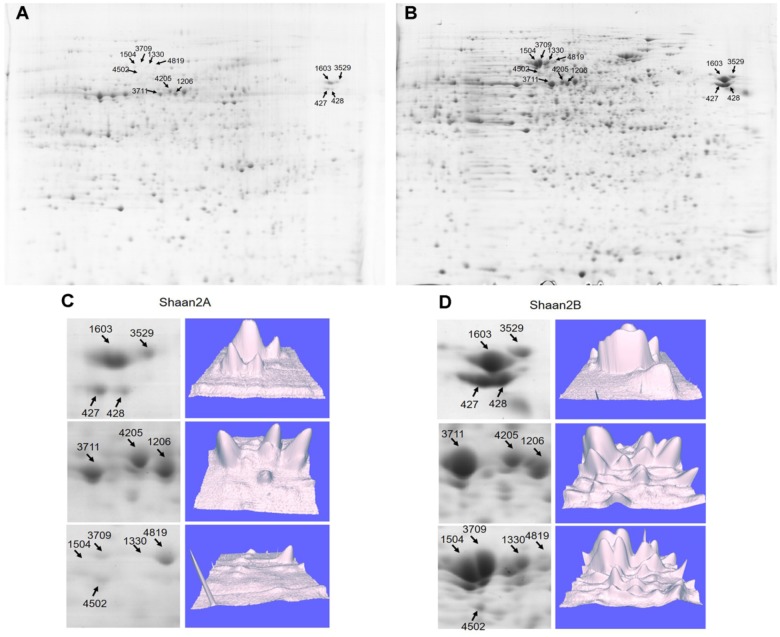
Representative images of DEPs between Shaan2A and Shaan2B. **(A)** Shaan2A 2-DE images. **(B)** Shaan2B 2-DE images. **(C)** Enlarged area of 2-DE gel and 3D images of Shaan2A. **(D)** Enlarged area of 2-DE gel and 3D images of Shaan2B.

To detect DEPs between Shaan2A and Shaan2B, we conducted 2-DE with three biological triplicates using total protein from anthers of Shaan2A and Shaan2B. Approximately 1,000 protein spots were detected (Figure 6A,B). DEPs exceeding the least significant difference (*p* < 0.05) and showing more than twofold change in abundance were chosen for MALDITOF-MS-MS analysis.

**Table 2:** Identification and relative expression level of DEPs.

Spot no.	Identified protein	Arabidopsis homolog	Mr	Score	Relative expression level	
					Shaan2A	Shaan2B
**Amino acid metabolism**						
115	Glutathione *S*-transferase	AT2G30860	22,630	130	4,671.87	500.30
2535	*S*-Adenosylmethionine synthase 2	AT4G01850	43,627	539	189.00	3,546.70
3529	*S*-Adenosylmethionine synthase 2	AT3G17390	43,309	300	206.40	600.83
8302	Spermidine synthase 1; SPDSY 1	AT1G23820	36,986	290	71.60	272.43
8311	Spermidine synthase 1; SPDSY 1	AT1G23820	36,986	572	471.43	969.47
1106	Glutathione *S*-transferase	AT2G30860	59,422	241	6,979.03	1,108.27
**Biosynthesis of other secondary metabolites**						
1603	PREDICTED: hydroxycinnamoyl-coenzyme A shikimate/quinate hydroxycinnamoyltransferase-like	AT5G48930	47,658	47	737.73	3,454.70
**Carbohydrate metabolism**						
329	Similar to uridine diphosphate glucose epimerase; F8M12.10	AT4G10960	38,804	66	305.97	2,293.53
423	Fructose-bisphosphate aldolase, class I	AT3G52930	38,858	405	0.00	277.00
427	Unnamed protein product	AT1G13440	32,088	430	618.40	1,761.33
428	Acetyl-CoA C-acetyltransferase	AT5G48230	51,531	285	557.00	2,071.93
2325	Putative aldose 1-epimerase	AT3G17940	35,355	60	148.57	1,120.93
2426	Glutamate-ammonia ligase (EC 6.3.1.2), cytosolic (clone lambdaAtgskb6) – Arabidopsis thaliana	AT3G17820	40,932	140	2,440.57	5,153.13
2502	Ribulose-1,5-bisphosphate carboxylase/oxygenase large subunit	ATCG00490	53,282	555	22,765.10	3,667.27
3223	Stromal ascorbate peroxidase	AT4G08390	38,737	272	108.83	504.83
3611	Inositol-3-phosphate synthase	AT2G22240	56,487	129	64.03	488.60
3711	ATA27	AT1G75940	62,096	77	300.67	3,023.13
4101	Nuclear-encoded chloroplast stromal cyclophilin CYP20-3 (also known as ROC4)	AT3G62030	26,725	363	2,615.93	621.03
6406	Glutamine synthetase, chloroplastic	AT5G35630	47,714	626	274.30	753.23
**Cellular processes**						
5423	Actin-3	AT3G53750	42,022	538	462.07	2,382.53
**Energy metabolism**						
215	Predicted NADH dehydrogenase 24 kD subunit	AT4G02580	27,564	82	320.23	2,120.83
1206	*S*-Formylglutathione hydrolase	AT2G41530	31,921	71	455.93	968.10
3104	Quinone reductase family protein	AT4G27270	21,778	175	46.90	247.10
4117	Chlorophyll a-b binding protein 6A, chloroplastic	AT3G54890	26,786	276	3,425.50	544.90
4205	*O*-Acetylserine (thiol)-lyase	AT4G14880	33,995	110	137.63	429.27
6113	Oxygen-evolving enhancer protein 2, chloroplastic	AT1G06680	26,712	268	4,478.83	1,069.63
6236	V-type proton-ATPase	AT4G11150	26,282	300	2,855.87	9.40
6523	ATP synthase beta subunit	ATCG00480	35,998	210	1,494.23	275.73
6620	Nucleotide-binding subunit of vacuolar ATPase	AT1G76030	54,819	335	1,170.23	272.30
7202	Photosystem II subunit O-2	AT3G50820	35,323	764	9,246.37	2,165.53
**Genetic information processing**						
6310	60S acidic ribosomal protein PO	AT3G09200	33,775	129	395.73	2,349.70
219	Multicatalytic endopeptidase	AT5G40580	23,960	162	197.47	669.53
1733	F23N19.10	AT1G62740	67,625	93	90.47	420.27
1818	Unnamed protein product	AT1G56070	94,734	314	129.30	999.50
2112	AT3g62030	AT3G62030	28,532	102	3,685.03	399.87
3034	SERINE CARBOXYPEPTIDASE II-like protein	AT4G30810	47,658	158	162.33	417.30
3421	Carboxypeptidase Y-like protein	AT3G10410	60,386	139	1,789.60	4,428.77
3627	ATP-dependent Clp protease ATP-binding subunit ClpC	AT5G50920	103,616	618	137.60	712.53
4303	Thiol protease isoform B, partial	AT4G39090	35,687	54	203.93	839.57
4502	Translational initiation factor 4A-1	AT3G13920	46,960	93	56.43	226.30
4530	Chloroplast elongation factor tub	AT4G20360	51,868	763	288.33	836.20
4819	Chaperone protein ClpC, chloroplastic	AT5G50920	102,818	307	223.40	466.57
5601	Hypothetical protein ARALYDRAFT_478789	AT3G13860	60,881	289	240.83	563.97
6206	20S proteasome subunit PAF1	AT5G42790	30,543	618	131.47	363.77
6208	20S proteasome subunit PAF1	AT5G42790	30,543	303	129.37	317.57
7125	Immunophilin	AT5G48580	17,790	118	312.90	631.67
8001	Hypothetical protein ARALYDRAFT_897824	AT3G15360	21,277	185	756.80	1,944.37
8602	Protein disulfide-isomerase A1	AT1G21750	55,852	165	695.37	2,317.87
8604	Protein disulfide-isomerase A1	AT1G21750	55,852	93	201.87	419.27
**Glycan biosynthesis and metabolism**						
1706	Glycosyl hydrolase family 38 protein	AT5G13980	11,6351	103	327.43	53.80
4304	Reversibly glycosylated polypeptide 1	AT3G02230	41,116	371	1,145.77	2,755.63
7322	Alpha-1,4-glucan-protein synthase family protein	AT5G16510	39,017	293	570.67	1,520.07
**Lipid metabolism**						
1306	Enoyl-[acyl-carrier protein] reductase	AT2G05990	40,944	438	387.17	1,824.67
1504	3-Ketoacyl-acyl carrier protein synthase I	AT5G46290	50,890	220	127.87	1,155.43
**Nucleotide metabolism**						
7412	Adenosine kinase 2	AT5G03300	38,221	160	37.03	209.77
**Signal transduction**						
3709	Auxin responsive-like protein	AT5G13370	65,180	85	212.30	1,286.10
**Unclassified**						
1205	Cysteine proteinase inhibitor	AT3G12490	22,988	162	372.03	952.07
1330	Annexin E1	AT5G65020	36,109	357	95.03	824.60
1416	Chalcone synthase 3 protein	AT1G02050	43,493	239	512.73	1,551.23
2108	Superoxide dismutase	AT4G25100	23,791	323	9,351.10	1,307.23
3720	Os09g0511700	AT1G02850	30,364	46	156.40	1,057.57
4424	Chalcone synthase 2 protein	AT4G00040	43,246	444	83.77	285.20
6317	Uncharacterized protein	AT5G08540	38,745	118	707.57	2,596.87
8225	Thaumatin-like protein	AT1G75050	26,267	116	406.47	1,725.40
3025	Acyltransferase homolog	AT5G23940	50,231	54	0.00	100.93
8120	Conserved hypothetical protein	AT5G48480	16,993	49	832.07	1,887.43

Compared with Shaan2B, 13 and 53 DEPs showed increased and decreased expression, respectively, in Shaan2A (Table 2). These DEPs were grouped into 11 categories, including “genetic information processing” (28.8%), “carbohydrate metabolism” (18.2%), “energy metabolism” (15.2%), and “amino acid metabolism” (9.1%) (Table 2). The enlarged 3D area of the 2-DE gel of some representative DEPs is shown in Figure 6C,D. For example, hydroxycinnamoyl-CoA: shikimate hydroxycinnamoyl transferase (spot 1603; HCT, AT5G48930) is related to the accumulation of flavonoids and in turn represses auxin transport to inhibit plant growth (Li et al., 2010). Methionine adenosyltransferase 4 (spot 3529; MAT4, AT3G17390) plays an important role in *S*-adenosyl-Met (SAM) production and plant development (Meng et al., 2018). β-Ketoacyl-[acyl carrier protein] synthase I (spot 1504; KASI, AT5G46290) is important for fatty acid synthesis and plays a key role in embryo development and chloroplast division (Wu and Xue, 2010). More details are shown in Table 2, and all of these proteins were down-regulated in Shaan2A.

**FIGURE 7:**
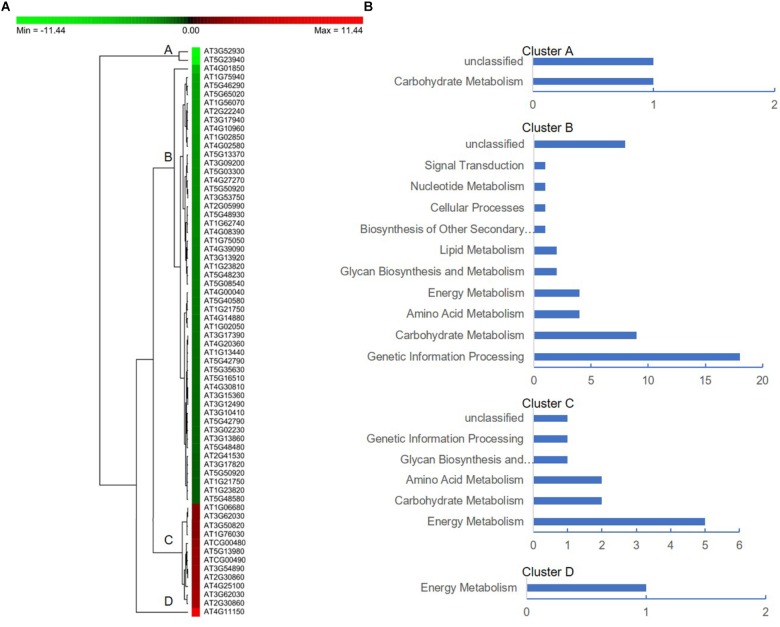
Protein expression profiles of the DEPs between Shaan2A and Shaan2B. **(A)** Hierarchical clustering of proteins showing quantitative changes in expression. **(B)** Protein functional classification of four clusters.

To further facilitate the biological interpretation of the identified DEPs, we conducted hierarchical clustering analysis of the DEPs showing quantitative changes in expression. We identified four clusters of DEPs (Figure 7A) with dramatic changes in expression patterns between fertile and sterile anthers. Cluster A contained two proteins, one belonging to the “carbohydrate metabolism” group and the other unknown (Figure 7B), and both were dramatically down-regulated in Shaan2A compared with Shaan2B. Cluster B, the largest cluster, included 51 proteins down-regulated in Shaan2A, with the “genetic information processing” and “carbohydrate metabolism” categories dominating (Figure 7B); for example, acetyl-CoA C-acetyltransferase protein (spot 428; AT5G48230) and adenosine kinase 2 (spot 7412; AT5G03300). Cluster C proteins showed increased expression in Shaan2A compared with Shaan2B, and included 12 proteins with “energy metabolism” as the dominant classification (Figure 7B); for instance, ATP synthase beta subunit (spot 6523; ATCG00480) and ribulose-1,5-bisphosphate carboxylase/oxygenase large subunit (spot 2502; ATCG00490). Cluster D contained only one protein with high expression in Shaan2A and low expression in Shaan2B (Figure 7B). This was a V-type proton-ATPase protein related to “energy metabolism.”

### Correlation Analysis Between DEGs and DEPs

*B. napus* is an allotetraploid plant, and its genome contains many repeats and homologous sequences (Chalhoub et al., 2014). The 66 DEPs identified in the proteomic analysis corresponded to 344 genes in *B. napus*. We conducted correlation analysis between DEGs and DEPs at both the YB and SA stage. DEPs and DEGs were divided into four groups according to their expression in Shaan2A compared to Shaan2B (Supplementary Table S4). In Group I, 43 and 115 DEGs showed the same trend in expression as their corresponding DEPs at the YB and SA stage, respectively. For example, *actin 3* (*AT3G53750*) and *chalcone and stilbene synthase family protein* (*AT4G00040*), which are associated with protein processing in the endoplasmic reticulum, and *SAM*. In Group II, 21 and 24 DEGs showed the opposite trend in expression from their corresponding DEPs at the YB and SA stage, respectively, such as *MAT4* (*AT3G17390*) and *GLN1.3* (*AT3G17820*), associated with amino acid and carbohydrate metabolism, respectively. In Group III, the corresponding genes of 280 and 205 DEPs at the YB and SA stage, respectively, were not differentially expressed. In Group IV, the genes corresponding to 33 DEPs at the YB stage and 10 DEPs at the SA stage did not show differential expression in the transcriptomic analysis. These results showed that both positive and negative correlations exist between the mRNA and protein expression profiles at different stages, indicating that there might be a complex post-transcriptional regulatory network in Shaan2A male sterility.

### Gene Ontology (GO) Analysis of DEGsand DEPs

**FIGURE 8:**
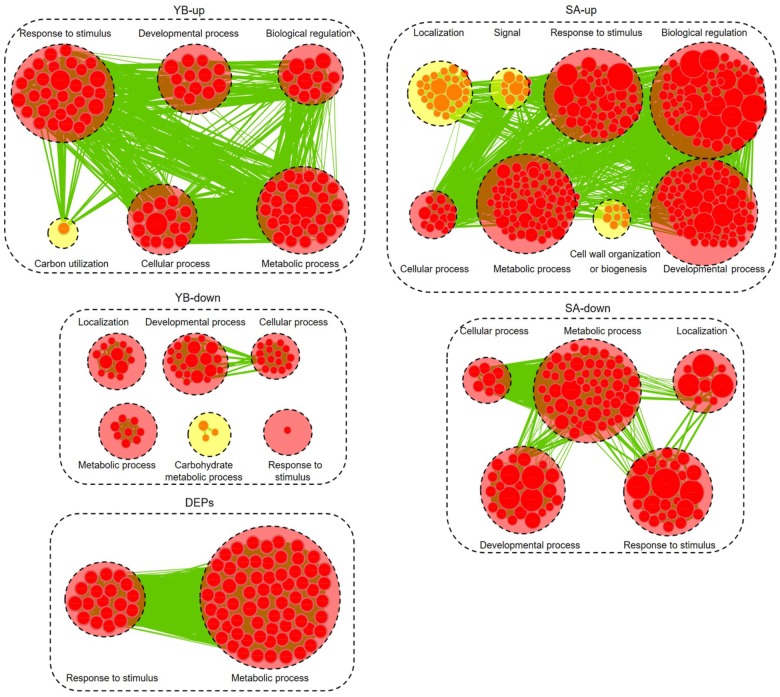
Biological process analysis of DEGs and DEPs. GO modules enriched by up- and down-regulated DEGs and DEPs visualized using Cytoscape EnrichmentMap. Yellow and red circles show different and common biological processes between up- and down-regulated DEGs, respectively. The related GO categories are circled with a black dashed ring.

We further analyzed the up- and down-regulated DEGs (| log_2_ ratio| > 1) at the YB and SA stages and total DEPs between Shaan2A and Shaan2B using Cytoscape BiNGO and visualized the functional enrichment with Cytoscape EnrichmentMap. As shown in Figure 8, YB-up, YB-down, SA-up, SA-down, and DEPs generated 102, 59, 300, 117, and 85 nodes, respectively. These nodes were classified into different categories. Interestingly, the common GO terms among these stages were “response to stimulus” and “metabolic process,” which might largely be due to the toxic action of the sterility gene in mitochondria disrupting the balance of metabolic processes in Shaan2A. Very importantly, nine categories associated with anther and pollen development were enriched among down-regulated DEGs at the YB stage: “androecium development,” “anther development,” “microsporogenesis,” “pollen development,” “pollen exine formation,” “pollen wall assembly,” “sporopollenin biosynthetic process,” “stamen development,” and “tapetal layer development.” This indicates that anther and pollen development were disordered at the YB stage in Shaan2A, consistent with the cytological observations mentioned above. Expression levels of the genes in these GO categories at the YB stage are given in Supplementary Table S5. For example, three copies of *CYP703A2* were remarkably down-regulated (*BnaA10g00600D*, log_2_ ratio = −13.53; *BnaC05g00670D*, log_2_ ratio = −13.30; *BnaCnng08630D*, log_2_ ratio = −10.29); this gene is expressed in developing anthers, and knockout lines display a partial male sterile phenotype in Arabidopsis (Morant et al., 2007). Two copies of *LESS ADHESIVE POLLEN 6* (*LAP6*) were also significantly down-regulated (*BnaA04g21720D*, log_2_ ratio = −9.50; *BnaC04g45570D*, log_2_ ratio = −12.63); this gene is transiently and specifically expressed in tapetal cells during pollen development in Arabidopsis, and the mutants show defective pollen exine (Kim et al., 2011).

### Transcription Factor Analysis

Transcription factors play key roles in multiple biological processes. We identified 349 up-regulated and 464 down-regulated TFs in Shaan2A compared to Shaan2B at the YB stage, and 867 up-regulated and 596 down-regulated TFs at the SA stage (*P* < 0.05, Supplementary Table S6). Most genes belonging to “bHLH,” “C2H2,” “MADS,” and “MYB” TF families were down-regulated in Shaan2A at the YB stage. Interestingly, 110 TFs were only expressed in Shaan2B at this stage. These included two copies of *DYT1* (a putative bHLH TF), predicted to play a key role in a putative regulatory network model for Arabidopsis anther development, and three copies of *MYB80*, the downstream gene of *DYT1* (Feng et al., 2012; Cui et al., 2016). Further, some TFs reported to be key regulators of anther differentiation were down-regulated at the YB stage.

### Genes Related to Early Anther Differentiation

During early anther differentiation, *SPL*, which is activated by *AG*, together with its down-stream genes, specifies archesporial cells in Arabidopsis before meiosis (Feng and Dickinson, 2007). The expression levels of 23 of these genes at the YB stage are shown in Supplementary Table S7, with 12 of them significantly differentially expressed between Shaan2A and Shaan2B. Among these, three genes were slightly up-regulated in Shaan2A: *ER*, *GRF*, and *ERL2*. Interestingly, the eight down-regulated genes showed large log_2_ ratios for expression level; for example, two copies of *SPL* (*BnaA01g16350D*, log_2_ ratio = −2.72; *BnaC01g19500D*, log_2_ ratio = −2.66), *ROXY2* (*BnaA02g01990D*, log_2_ ratio = −4.72), four copies of *AMS* (*BnaA03g39400D*, log_2_ ratio = −13.00; *BnaA07g03340D*, log_2_ ratio = −9.00; *BnaC03g46740D*, log_2_ ratio = −10.33; *BnaC07g05950D*, log_2_ ratio = −11.92), and two copies of *MS1* were only expressed in Shaan2B. There were 11 genes with no significant difference in expression between Shaan2A and Shaan2B or no expression in either the sterile or maintainer lines, for instance, *AG*, *SPL8*, *TPD1*, and *SERK1/2*, indicating that they might not participate in Shaan2A CMS.

### Interaction Analysis for Candidate Genes

**FIGURE 9:**
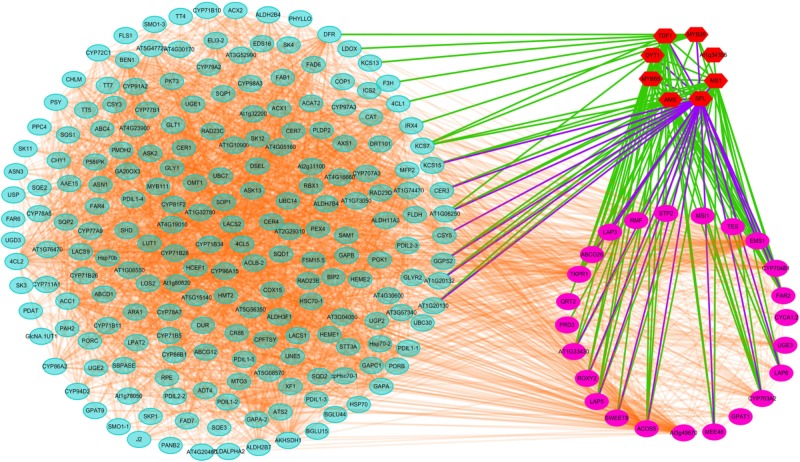
Interaction analysis of candidate proteins. Red nodes represent TFs related to early anther differentiation. Rose red nodes represent proteins in the nine GO categories of anther and pollen development. Light blue nodes represent proteins in the significantly enriched pathways identified by KEGG or MapMan analysis.

Transcriptomic and proteomic analyses suggested that CMS in Shaan2A is controlled by a complex regulatory network. To further elucidate this mechanism, we investigated known and predicted interactions among candidate proteins corresponding to our DEGs and DEPs; for example, TFs associated with anther differentiation and genes belonging to the anther and pollen development GO categories mentioned above. Protein–protein interactions based on Arabidopsis orthologs were identified using STRING 10.0 and visualized using Cytoscape 3.6.1, and an interaction network associated with anther development was constructed. As shown in Figure 9, this network contained 236 nodes. Interestingly, TFs down-regulated at the YB stage showed interactions with many other proteins. For instance, SPL interacted with 26 targets including MYB65, TDF1, ROXY2, AMS, and MS1, DYT1 interacted with 19 targets including ABCG26, LAP3, LAP5, AT1G33430, and CYP704B1, and MYB65 interacted with seven targets including SPL, AMS, MS1, and DYT1. Therefore, these TFs and their target proteins might form a complex network for regulating anther differentiation and pollen development in Shaan2A CMS.

## Discussion

In recent years, CMS has been widely studied by transcriptomic and proteomic analysis in different species, including onion (Yuan et al., 2018), cotton (Suzuki et al., 2013; Yang et al., 2018), maize (Li et al., 2017), and rapeseed (An et al., 2014; Du et al., 2016). These studies revealed that cotton CMS-D8 lines have reduced ability to eliminate ROS (Yang et al., 2018), secretion and translocation of sporopollenin precursors is significantly down-regulated in cabbage R2P2CMS (Xing et al., 2018), and significant DEGs in rapeseed SaNa-1A CMS and *pol* CMS mostly participate in protein synthesis and metabolic pathways (An et al., 2014; Du et al., 2016). In the present study, DEGs were enriched in “protein processing in endoplasmic reticulum,” “carbon metabolism,” and “fatty acid degradation” categories at the YB stage, and “starch and sucrose metabolism,” “porphyrin and chlorophyll metabolism,” and “carbon metabolism” categories at the SA stage. Moreover, DEPs indicated that functional processes of “genetic information processing,” “carbohydrate metabolism,” “energy metabolism,” and “amino acid metabolism” were disordered in Shaan2A.

### Shaan2A Is Defective in Early Anther Differentiation

Tapetum cells in a sterile onion CMS line begin to degrade from the tetrad stage and microspores start to abort, while degradation in its maintainer line is not observed until the microspore stage (Yuan et al., 2018). Fertile and sterile cotton anthers show differences at the meiotic stage, but no obvious variations before the stage when the microsporocytes and four anther wall cell layers are formed (Yang et al., 2018). In the C-type CMS of maize, no significant differences in microspores are observed between fertile and sterile plants until the mononuclear stage (Li et al., 2017). Although the pollen sac in the SaNa-1A CMS line forms normally, tapetum cells develop vacuolation at the microsporocyte and tetrad stages compared with the maintainer line (Du et al., 2016). In this study, the anther epidermis, endothecium, middle wall layers, tapetum, and sporogenous cells could not be clearly distinguished in Shaan2A by paraffin section observation (Figure 2). TEM analysis also revealed fewer mitochondria in the anther wall cells of Shaan2A than of Shaan2B, and the lamella structure of chloroplasts in Shaan2A anther wall cells was obviously abnormal (Figure 3). In contrast to Shaan2B, there was no difference between epidermis and tapetum cells in Shaan2A at the late stage of anther differentiation (Figure 3). Hence, we deduce that defective differentiation of anther wall cells and sporogenous cells in Shaan2A results in male sterility.

### Shaan2A Anther Cells Have Damaged Mitochondria

Cytoplasmic male sterility is a maternally inherited trait resulting from the interaction of a nuclear fertility restoring gene (*Rf*) and a mitochondrial CMS gene (Horn et al., 2014; Yamagishi and Bhat, 2014). A few CMS-associated genes have been reported, for example, *orf224* in *pol* CMS (L’Homme and Brown, 1993; Menassa et al., 1999) and *orf138* in *Ogu* CMS (Duroc et al., 2005). However, the sterility gene in the Shaan2A mitochondrial genome has not been identified. The mitochondrion is a crucial organelle for metabolic pathways such as respiratory electron transfer, ATP synthesis, and the TCA cycle (Reichert and Neupert, 2004; Logan, 2006). As well as the number of mitochondria in Shaan2A anthers being less than that in Shaan2B, most DEGs associated with the “mitochondrial electron transport/ATP synthesis pathway” and “TCA cycle” were obviously down-regulated at the YB and SA stages in Shaan2A anthers (Figure 5), including some important genes such as *Bnac04g22440D* (*CITRATE SYNTHASE 5*, log_2_ ratio = −7.69 at YB stage), *BnaC03g08060D* (*NAD(P)H DEHYDROGENASE B4*, log_2_ ratio = −5.55 at YB stage), and *BnaA05g17260D* (*AOX1a*, log_2_ ratio = −2.34 at YB stage). Additionally, 15.2% DEPs were grouped into the “energy metabolism” functional category. It is interesting that AT4G11150 (a V-type proton-ATPase) and AT1G76030 (a nucleotide-binding subunit of vacuolar ATPase) were both up-regulated in Shaan2A anthers compared to Shaan2B anthers; if defective mitochondria in Shaan2A do not produce enough energy to meet the needs for rapid anther growth, these ATPases might be up-regulated to rescue the energy metabolism in the sterile anthers. As the sterility gene in Shaan2A is unknown, the mechanism by which it functions and induces a series of response in mitochondria will need further study.

### Shaan2A Anther Cells Have Injured Chloroplasts

The abundance of starch in chloroplasts is necessary for microspore development and is a crucial feature of fertile pollen (Du et al., 2016). Most studies of male sterility in plants have identified “carbon metabolism” as disordered (Cheng et al., 2013; Li et al., 2015, 2016; Du et al., 2016; Liu et al., 2018; Yuan et al., 2018). Interestingly, the lamella structure of chloroplasts in Shaan2A anther wall cells was obviously abnormal. Most DEGs involved in “carbon metabolism,” “light reactions,” “photorespiration,” and “Calvin cycle” at the YB and SA stages (Figure 5 and Supplementary Table S3) and in “starch and sucrose metabolism” and “porphyrin and chlorophyll metabolism” at the SA stage were up-regulated (Supplementary Table S2). However, proteomic analysis revealed that the 12 DEPs grouped into “carbohydrate metabolism” were mostly down-regulated, except for ATCG00490, (ribulose-1,5-bisphosphate carboxylase/oxygenase large subunit, encoded by a chloroplast gene) and AT3G62030 (a nuclear-encoded chloroplast stromal cyclophilin CYP20-3, also known as ROC4). Despite many carbon metabolism related pathways being identified by up-regulated genes, chloroplasts of Shaan2A contained fewer starch grains than those in Shaan2B (Figure 3). Therefore, we speculate that high expression of many genes related to carbon metabolism is triggered by the injured chloroplast structure in Shaan2A. However, this up-regulation cannot balance the carbon metabolism in cells, which might be affected by the sterility gene. The direct or indirect mechanism by which the sterility gene impacts chloroplasts requires further study.

### Genes Involved in Early Anther Differentiation Are Down-Regulated at the YB Stage

*SPL* functions downstream of *AG*, specifying the identity, position, and number of archesporial cells in the anther L2 layer (Schiefthaler et al., 1999; Yang et al., 1999). We identified two copies of *SPL* that were down-regulated in Shaan2A compared with Shaan2B, while three copies of *AG* were not significantly expressed (Supplementary Table S7). We therefore presume that *SPL* is the first gene repressed by the sterility gene in the mitochondrion. Furthermore, SPL was predicted to interact with 26 targets (Figure 9). These include TFs such as MYB65, ROXY2, AMS, MS1, and DYT1, which were also obviously down-regulated in Shaan2A (Supplementary Table S7). In Arabidopsis, *MYB33* and *MYB65* share high sequence similarity. The *myb33 myb65* double mutant has a deficiency in anther development, with hypertrophied tapetum at the pollen mother cell stage and aborted microspores before meiosis; the single mutant shows no phenotype, implying that *MYB65* and *MYB33* are functionally redundant (Millar, 2005). In *B. napus*, we only identified putative orthologs of *AtMYB65*; however, three copies of *BnMYB65* were significantly down-regulated. *ROXY1* and *ROXY2* together control anther development in Arabidopsis, and single mutants produce normal anthers; however, double mutants are sterile owing to defects in early lobe differentiation (Xing and Zachgo, 2008). In the present study, two copies of *ROXY2* showed significant down-regulation in Shaan2A compared with Shaan2B (Supplementary Table S7). In addition, four copies of *AMS*, two copies of *MS1*, and three copies of *DYT1* exhibited large log_2_ ratios of down-regulation in Shaan2A (Supplementary Table S7); all of these are located downstream of *SPL* and essential for anther and pollen development in Arabidopsis (Wilson et al., 2001; Feng and Dickinson, 2007; Feng et al., 2012; Ferguson et al., 2017).

SPL was also predicted to interact with ABCG26, MEE48, LAP5, LAP6, ACOS5, EMS1, and other proteins (Figure 9). In Arabidopsis, ABCG26 is an ATP binding cassette (ABC) transporter localized to the plasma membrane and endoplasmic reticulum that mediates trafficking of hydroxycinnamoyl spermidines and polyketides needed for sporopollenin formation (Quilichini et al., 2010, 2014). *Maternal Effect Embryo Arrest 48* (*MEE48*) is related to morphogenesis and development (Chakraborty et al., 2015). LAP5 and LAP6 catalyze condensation of malonyl-CoA units with CoA ester starter molecules to generate various natural products, and are required for sporopollenin biosynthesis and pollen development (Kim et al., 2011). The *acyl-CoA synthetase 5* (*ACOS5*) gene encodes a fatty acyl-CoA synthetase that plays a key role in sporopollenin biosynthesis and exine formation (de Azevedo Souza et al., 2009). *EMS1* encodes a putative LRR receptor protein kinase (LRR-RPK) controlling reproductive cell fates in anthers (Canales et al., 2002; Zhao et al., 2002). Interestingly, in *B. napus*, six copies of *MEE48*, seven copies of *LAP5*, two copies of *LAP6*, and two copies of *ACOS5* showed high log_2_ ratios of down-regulation in Shaan2A or were only expressed in Shaan2B, and two copies of *EMS1* also displayed lower expression in Shaan2A than in Shaan2B (Supplementary Tables S5, S7). These genes were also predicted to interact with many other genes (Figure 9). Taken together, we propose that after the male sterility signal from the mitochondrion is transferred to the nucleus, it initiates suppression of *SPL* expression resulting in down-regulation of its downstream genes, which form a huge and complicated regulatory network for anther differentiation in Shaan2A. However, the mechanism underlying this network requires further research.

## Data Availability

Transcriptomic raw reads were submitted to the SRA (Sequence Read Archive of NCBI) database with accession number PRJNA502996.

## Author Contributions

ML and HW conceived and designed the experiments. LN and ZL performed the experiments and analyzed the data. DL provided the Shaan2A and Shaan2B materials. YL, WZ, HC, and LM helped to analyze the data. LN and ML wrote the manuscript. All authors read and approved the final manuscript.

## Conflict of Interest Statement

The authors declare that the research was conducted in the absence of any commercial or financial relationships that could be construed as a potential conflict of interest.

## Footnotes

1 http://www.genome.jp/kegg/

2 https://string-db.org/

## Supplementary Material

FIGURE S1 TEM observation of anther wall cells in Shaan2A and Shaan2B. **(A)** Overview of anther wall cells in 1 mm buds of Shaan2A. **(B)** Overview of anther wall cells in 2 mm buds of Shaan2A. **(C)** Overview of anther wall cells in 3 mm buds of Shaan2A. **(D)** Overview of anther wall cells in 1 mm buds of Shaan2B. **(E)** Overview of anther wall cells in 2 mm buds of Shaan2B. **(F)** Overview of anther wall cells in 3 mm buds of Shaan2B. E, epidermis; En, endothecium; ML, middle layer; T, tapetum; MMC, microspore mother cells.

FIGURE S2 Validation of DEGs by qRT-PCR. **(A)** Result from qRT-PCR. **(B)** Result from RNA-seq. Numbers are log_2_(X)-normalized ratio values. Red represents higher gene expression levels. Green corresponds to lower gene expression levels. Blocks without a numerical value indicate that gene expression was not significantly detected by RNA-seq.

TABLE S1 Primers used in qRT-PCR.

TABLE S2 Enriched KEGG pathways identified from DEGs at the YB and SA stages. Pathways were considered enriched at corrected *P* < 0.05.

TABLE S3 Detailed information on metabolic pathways from MapMan analysis.

TABLE S4 Correlation analysis between DEGs and DEPs. Y represents positive correlation between DEGs and DEPs. N represents negative correlation between DEGs and DEPs.

TABLE S5 Expression levels of genes in the nine GO categories associated with anther and pollen development at the YB stage.

TABLE S6 Transcription factors identified at the YB and SA stages.

TABLE S7 Expression levels of genes related to early anther differentiation at the YB stage.
