# Supplementary figures and images for: Transcriptomic and Proteomic Analysis of Shaan2A Cytoplasmic Male Sterility and Its Maintainer Line in Brassica napus
Source: Front Plant Sci. 2019 Mar 4;10:252. doi: 10.3389/fpls.2019.00252 (PMC6409359; doi:10.3389/fpls.2019.00252)

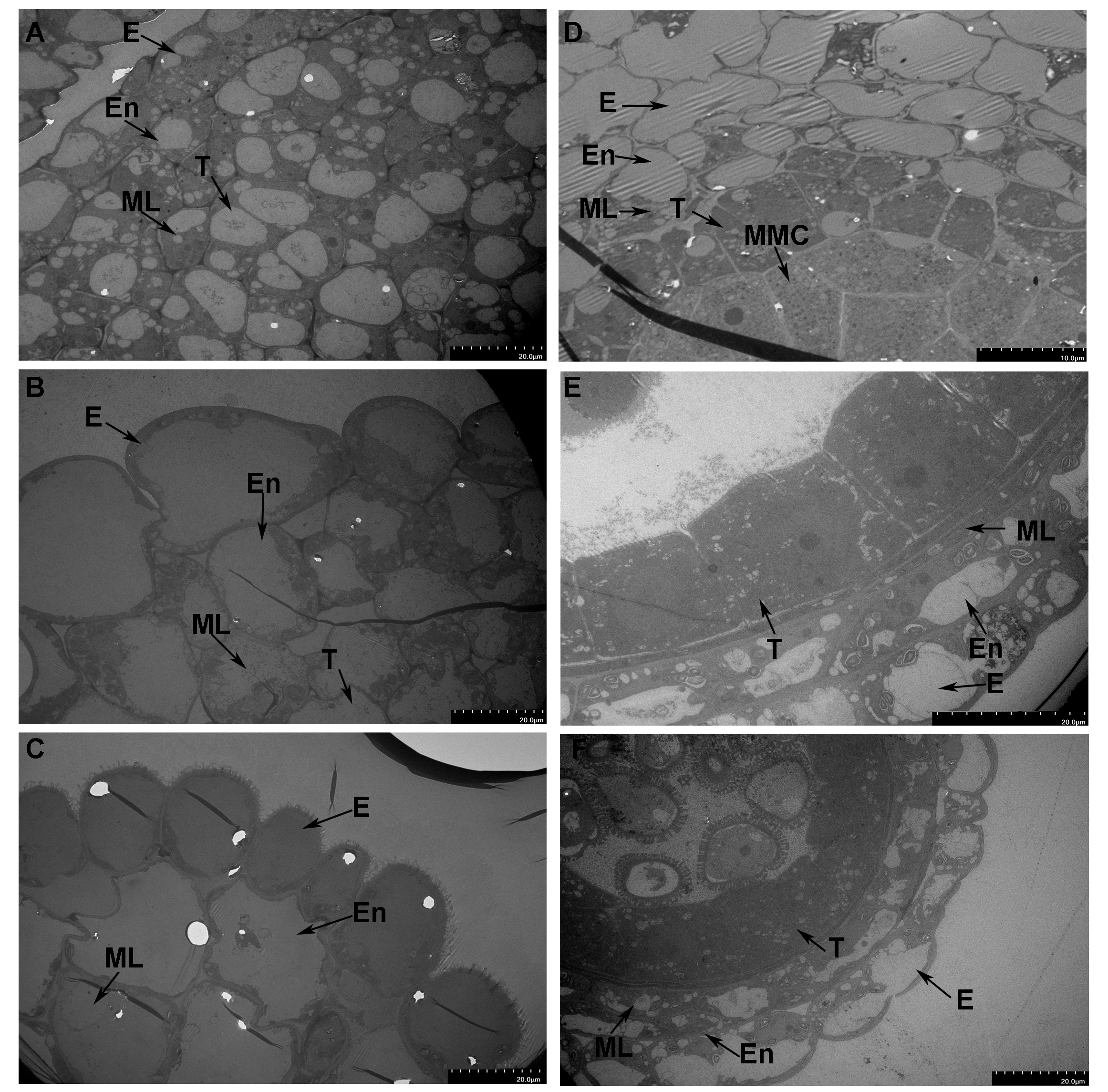

Supplement: FIGURE S1 — TEM observation of anther wall cells in Shaan2A and Shaan2B. (A) Overview of anther wall cells in 1 mm buds of Shaan2A. (B) Overview of anther wall cells in 2 mm buds of Shaan2A. (C) Overview of anther wall cells in 3 mm buds of Shaan2A. (D) Overview of anther wall cells in 1 mm buds of Shaan2B. (E) Overview of anther wall cells in 2 mm buds of Shaan2B. (F) Overview of anther wall cells in 3 mm buds of Shaan2B. E, epidermis; En, endothecium; ML, middle layer; T, tapetum; MMC, microspore mother cells. [file Image_1.JPEG]

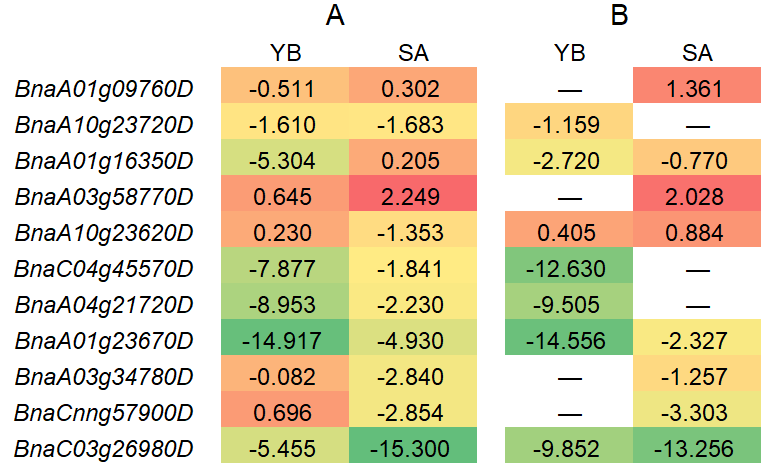

Supplement: FIGURE S2 — Validation of DEGs by qRT-PCR. (A) Result from qRT-PCR. (B) Result from RNA-seq. Numbers are log2(X)-normalized ratio values. Red represents higher gene expression levels. Green corresponds to lower gene expression levels. Blocks without a numerical value indicate that gene expression was not significantly detected by RNA-seq. [file Image_2.PNG]
